# Dual-Crosslinking of Gelatin-Based Hydrogels: Promising Compositions for a 3D Printed Organotypic Bone Model

**DOI:** 10.3390/bioengineering10060704

**Published:** 2023-06-09

**Authors:** Ahmer Shehzad, Fariza Mukasheva, Muhammad Moazzam, Dana Sultanova, Birzhan Abdikhan, Alexander Trifonov, Dana Akilbekova

**Affiliations:** Department of Chemical Engineering, School of Engineering and Digital Sciences, Nazarbayev University, Astana 010000, Kazakhstan

**Keywords:** 3D bioprinting, hydrogel scaffolds, bone tissue engineering, dual crosslinking, gelatin

## Abstract

Gelatin-based hydrogels have emerged as a popular scaffold material for tissue engineering applications. The introduction of variable crosslinking methods has shown promise for fabricating stable cell-laden scaffolds. In this work, we examine promising composite biopolymer-based inks for extrusion-based 3D bioprinting, using a dual crosslinking approach. A combination of carefully selected printable hydrogel ink compositions and the use of photoinduced covalent and ionic crosslinking mechanisms allows for the fabrication of scaffolds of high accuracy and low cytotoxicity, resulting in unimpeded cell proliferation, extracellular matrix deposition, and mineralization. Three selected bioink compositions were characterized and the respective cell-laden scaffolds were bioprinted. Temporal stability, morphology, swelling, and mechanical properties of the scaffolds were thoroughly studied and the biocompatibility of the constructs was assessed using rat mesenchymal stem cells while focusing on osteogenesis. Experimental results showed that the composition of 1% alginate, 4% gelatin, and 5% (*w*/*v*) gelatine methacrylate, was found to be optimal among the examined, with shape fidelity of 88%, large cell spreading area and cell viability at around 100% after 14 days. The large pore diameters that exceed 100 µm, and highly interconnected scaffold morphology, make these hydrogels extremely potent in bone tissue engineering and bone organoid fabrication.

## 1. Introduction

Tissue engineering emerges as the platform for the development of artificial tissues and organs, which will not only help to cover the shortage of organ transplantation in the world but also provides the best alternative to in vivo animal testing [1]. In case of injury, bone regeneration follows the activation of the different growth factors and cytokines that causes osteoprogenitor cells to differentiate into osteoblast [2]. Yet, for congenital bone diseases such as osteoporosis and rheumatoid arthritis, the bone repair mechanism is more complicated and requires longer periods, frequently with less satisfactory healing results. Clinical solutions such as allografts, xenografts, and autografts are considered acceptable alternatives for functional recovery and bone regeneration applications. However, occasionally, these face failures in the form of transplant host rejection with a higher chance of disease transmission [3,4,5]. At the same time, the 3Rs principles referred to as reduction, replacement, and refinement, formulated by Russel and Burch [6], were developed to avoid, and ultimately, eliminate animal use for scientific experimental purposes. Although tissue replacement is widely appraised for bone repair treatments [7], the development of experimental organotypic in vitro models for process investigation in close-to-real conditions can significantly increase the success rate in existing treatments as well as progress in alternative methods. To develop such models, mimicking tissue/organ functionality, biomaterials should grant both mechanical strength and biocompatibility, providing adequate morphology and room for cell proliferation and formation of extracellular matrix (ECM). Natural biopolymers, in this context, have a great potential to satisfy these criteria, providing a close-to-natural chemical environment and highly porous morphology, allowing regulation of tissue formation parameters and monitoring cell behavior and tissue growth phases [8].

Moving from 2D to 3D cultures is a crucial step when replicating natural tissue. The introduction of higher complexity of fabrication and handling is justified by higher biological relevance and real-life applications. In the field of bone tissue engineering and organoid modeling, 3D bioprinting has gained a sizable rise due to the significant benefits demonstrated by numerous studies. 3D bioprinting allows for printing biomaterial-containing (e.g., cells, growth factors) scaffolds of desired geometries accurately, using a layer-by-layer bioink deposition [9]. Furthermore, bioprinting not only eliminates the need for traditionally used cell seeding procedures but also creates a homogeneous 3D environment. The layer-by-layer deposition of cell-laden biomaterial was demonstrated to advance the proper distribution of cells, which improves proliferation and contributes to the development of ECM components [10]. Control over the bioink composition and printing conditions helps to create scaffolds with controllable porosity for improved cell proliferation, supporting associated processes such as gas exchange, nutrient supply, and vascularization [11,12], and further mineralization of cell components at late stages [13,14].

Although 3D bioprinting technology allowed significant progress in tissue engineering, the formulation of bioinks with appropriate physical and biological properties and cytocompatible gelation mechanisms presents substantial challenges [15]. Typically, extrusion-based bioprinting relies on hydrogels originating from natural biopolymers such as gelatin (Gel), alginate (Alg), hyaluronic acid, collagen, fibrin, and others [16,17]. The suitability of inks for 3D bioprinting depends on their viscoelastic properties and cytocompatibility, as well as post-production characteristics such as shape fidelity and mechanical stability [18,19]. The latter is usually achieved and controlled by chemical crosslinking of biopolymers via condensation or radical polymerization, which facilitates the robustness and temporal stability of the scaffold [20].

Gelatin is a bioactive thermo-responsive protein and is widely used in tissue engineering due to its similarity to collagen. Furthermore, gelatin is an excellent biocompatible material that possesses cell adhesion domains and has superior viscoelastic properties. Gelation below 35 °C makes it ideal for extrusion-based 3D printing [21]. The low mechanical modulus and the fast degradation of gelatin can be improved by substituting part of its amino groups with methacryloyl units, revealing gelatin methacrylate (GelMA) building blocks [22,23,24]. GelMA lacks the same viscoelastic properties as gelatin, yet can provide the base for robust UV-induced chemical crosslinking and grant extended stability to the material. Doping of methacrylated precursor with photoinitiators such as 2-hydroxy-4′(hydroxyethoxy)-2-methylpropiophenone (irgacure-2959) or lithium phenyl-2,4,6-trimethylbenzoyilphosphinate (LAP) is widely used for UV initiated hydrogelation of 3D printed scaffolds. The irradiation time, the photoinitiator concentration, and the degree of functionalization (DoF) of the precursor mixture are the main parameters that allow tuning the physical properties and morphology of GelMA-containing hydrogels. However, an increase in photoinitiator concentrations leads to excessive hardness of the scaffold, insufficient swelling, reduced pore diameter, and low degradability [25,26,27]. Additionally, photoinitiator concentrations above 5% *w*/*v* were clearly shown to harm the cytocompatibility and long-term cellular viability, whereas lower concentrations might result in low crosslinking efficacy, and thus, reduced temporal stability of the scaffolds [28].

A combination of biopolymers can often provide a suitable solution for scaffold production, based on the characteristics and functionality of the components. Several attempts have been made to demonstrate GelMA-based bioprinted scaffolds for bone regeneration. Recently, Yin et al. tested 5%/8% Gelatin/GelMA hydrogel and demonstrated excellent bone marrow mesenchymal stem cell viability with high printing resolution. However, the resulting 3D-printed scaffolds were unable to address reasonable biodegradability to support osteogenic differentiation, compulsory for extracellular matrix formation in bone regeneration [29]. Cidonio et al. bioprinted laponite-GelMA nanocomposite to support the osteogenic differentiation, yet faced a low proliferation degree of human bone marrow stromal cells (HBMSC), reporting 75% cell viability after 21 days as compared to 95% viability using a sole GelMA precursor [30]. Liu et al. demonstrated good osteogenic properties by printing GelMA/struvite composite hydrogel with human dental pulp stem cells and showed excellent cell viability, yet reduced scaffold stability lasting for seven days only [31]. One of the studies conducted by Goto et al. demonstrated enhanced osteogenic differentiation in GelMA-riboflavin hydrogel encapsulated with mature osteoblast KUSA-A1, facing the same problem of low temporal stability and exhibiting no calcium deposition in the extracellular matrix as required for collagen formation [32]. Tavares et al. achieved osteogenic development in a bioprinted GelMA/silica composite with mesenchymal stem cells for up to 21 days, yet with no sizable calcium deposition or collagen synthesis [33]. Rastin et al. utilized human osteoblast-laden methyl cellulose/GelMA composite for scaffolds bioprinting, demonstrating 90% cell viability for up to 48 h, yet a bone model with calcium deposition and extracellular matrix development was not achieved [34].

It was suggested that the low temporal stability of the scaffolds, as well as compromises on cell viability, can be overcome using double crosslinking approaches, in which UV-induced radical crosslinking is combined with ionic bridging [35,36]. Whereas GelMA can be used as the main ingredient for chemical crosslinking, alginate (Alg) is ideal for ionic bridging in hydrogels due to its excellent biocompatibility, high gelation range, and reversible control over stiffness, which can be efficiently controlled via a Ca^2+^-assisted conjugation of alginate’s carboxylate functions [37,38].

A combination of covalent and ionic crosslinking mechanisms contributes to better energy dissipation for creating hydrogels of high toughness and elasticity. Whereas the mixture of GelMA/alginate is capable of maintaining both crosslinking mechanisms, its thermo-rheological characteristics are insufficient for high-fidelity 3D printing, yet can be well compensated by the addition of gelatin to achieve the desired viscoelastic properties.

Despite the progress achieved in the field of 3D bioprinting, employing the advantages of different natural and synthetic polymers, the challenge associated with the fabrication of hydrogel scaffolds of high shape fidelity, negligible cytotoxicity, and suitable degradation rate that supports rapid cell proliferation, ECM deposition, and mineralization as a set of factors are still unaddressed. This set of desirable characteristics is especially troublesome when it comes to the in vitro bone formation, in which morphology of large-diameter interconnected macropores (>100 µm) is highly preferable, as was experimentally shown for this type of tissue [39]. Fabrication of macropores above 100 µm diameter usually conflicts with high-accuracy printing and frequently leads to poor shape fidelity or structural collapse of the scaffold, impairing normal cell functioning and tissue development.

In the present work, we examine three promising alginate/gelatin/GelMA bioink formulations suitable for 3D bioprinting and examine the physical, morphological, and cytotoxic properties of the resulting scaffolds. The printing conditions were carefully optimized to obtain high printing accuracy and shape fidelity. Temporal stability was tuned using optimized precursor ratios and a dual crosslinking approach, combining photoinitiated covalent fixation and ionic bridging. Two out of three models showed excellent cell viability and biodegradability to support an effective osteogenic differentiation of cells and bone mineralization. The combination of demonstrated materials and curing process allows for the simple and cost-effective fabrication of cell-laden scaffolds with macroporous morphology, beneficial for bone tissue development and, therefore, a valuable addition to the library of methods and materials utilized in bone regeneration modeling and other in-vitro modeling applications.

## 2. Methods and Instrumentation

### 2.1. Materials

Gelatin from porcine skin (strength 300. Type A), sodium alginate (99% purity), lithium phenyl-2,4,6-trimethylbenzoylphosphinate (LAP), methacrylic anhydride (MA), sodium bicarbonate, phosphate-buffered saline (PBS), calcium chloride, live/dead viability assay kit, and cell culture reagents (Fetal bovine serum (FBS), 0.25% Trypsin EDTA, Dulbecco’s Modified Eagle Medium (DMEM), antibiotic-antimycotic solution), optimal cutting temperature compound (OCT), paraformaldehyde, sucrose, acetone, xylene, DPX mounting medium, Hematoxylin and Eosin, Alizarin Red S, ethanol were purchased from Sigma-Aldrich (Saint Louis, MO, USA). Rat mesenchymal stem cells (rMSCs) derived from the bone marrow of healthy animal donors were obtained from the National Center for Biotechnology, Astana, Kazakhstan.

### 2.2. GelMA Synthesis

The gelatin methacryloyl (GelMA) was synthesized by reacting methacrylate anhydrate with gelatin as previously reported, with minor changes. Briefly, 30 g of gelatin was dissolved in 300 mL of 100 mM PBS (pH = 7.2) at 50 °C by continuous stirring for 1 h to achieve a 10% *w*/*v* concentration.

To obtain a higher degree of functionalization (DoF), 10 *w*/*v*% methacrylic anhydride was added and the mixture was stirred at 400 rpm for 5 h at 50 °C. The supernatant was diluted three folds by the addition of the PBS and was dialyzed with 12–14 kDa cellulose membrane at 50 °C for 7 days and twice a day water exchange. The pH was adjusted to 7.4 using 1 M NaHCO_3_. The GelMA solution was frozen, lyophilized (Lyotrap Freeze Dryer, LTE Scientific, Oldham, UK), and stored at −20 °C for further use.

### 2.3. Degree of Functionalization

Methacrylation and degree of gelatin functionalization (DoF) were confirmed and assessed by recording nuclear magnetic resonance (NMR) proton spectra of unfunctionalized and methacrylated gelatin (GelMA), Figure S1 in Supplementary Materials. Here, 16 mg of each material (GelMA and gelatin) were separately dissolved in 1 mL of deuterium oxide D_2_O, transferred into two NMR tubes, and measured immediately [40]. The NMR spectra were recorded by 1H NMR spectrometry (JNM-ECA 500, JEOL, Tokyo, Japan) at a frequency of 400 MHz. MestreNova NMR software (version 6.0.2, Mestrelabs Research) was used for NMR data analysis. DoF (GelMA) was found to be 75.5% using Equation (1):

(1) $$DoF=\left(1-\frac{L_{GelMA}}{L_{Gelatin}}\right)\times 100\%$$

where *L_GelMA_* and *L_Gelatin_* are the areas under the peaks of lysine on 1H NMR of GelMA and gelatin, respectively, at 2.82 ppm.

### 2.4. Hygrogel Ink Preparation

Different ratios of gelatin and GelMA were mixed with 1% *w*/*v* alginate solution at 40 °C for 15 min to obtain three hydrogel ink compositions: I (1% Alg/4% Gel/5% GelMA), II (1% Alg/8% Gel/2.5% GelMA), and III (1% Alg/2% Gel/10% GelMA), where % stands for *w*/*v* fraction. The sterile LAP photoinitiator was added at a final concentration of 0.5% *w*/*v*, after optimization. The optimization criteria implied minimizing the photoinitiator concentration but leaving the ability for effective primary crosslinking, which allows for maintaining the scaffold’s shape after printing. All inks were prepared one day before printing and filtered with a 0.45 μm syringe filter before use.

### 2.5. Rheology

A rheometer (Anton Paar, MCR302, Graz, Austria) was used to test the hydrogel inks’ mechanical and viscoelastic properties. A 50 mm cone plate parallel geometry was used to measure the oscillatory test for the temperature-based dependency of the storage modulus G′ and loss modulus G″ ranging from 10 to 40 °C at the heating rate of 1.00 °C·min^−1^ with the constant frequency of 1 Hz and constant shear strain (γ) of 1%. Moreover, the dependency relationship between shear rate and viscosity was also examined in a rotation test at 15 °C with a shear rate between 0.1 and 100 s^−1^ in a flow curve analysis.

### 2.6. Cell Culture

Rat mesenchymal stem cells (rMSCs) (passage 2) were recovered and were expanded for 7 days in an expansion medium consisting of DMEM, 10% fetal bovine serum (FBS), 1% penicillin/streptomycin, 1% non-essential amino acid (NEAA), and 1 ng/mL basic fibroblast growth factor (bFGF). The medium was exchanged every three days. Cells were passaged using 0.25% trypsin EDTA for detachment. The osteogenic media containing DMEM with 10% FBS, 1% penicillin/streptomycin, and 1% NEAA, 50 μg/mL L-Ascorbic acid-2-phosphate sesquimagnesium salt hydrate, 100 nM dexamethasone, and 10 mM β-glycerophosphate was prepared to differentiate rMSCs to osteoblasts. 

### 2.7. 3D Bioprinting

BioX 3D printer (CELLINK, Goteborg, Sweden) was used to print all scaffolds using a temperature-controlled printhead and printing stage to ensure good printability conditions. The rMSCs at a concentration of 5 million cells/mL were centrifuged for 10 min at 4 °C, resuspended in 100 μL cell culture medium, and added to 1 mL of the hydrogel ink solutions. Prepared bioinks were loaded to the extrusion heads and placed in ice for 2 min. After gelation in the printhead, a lattice-rod model with dimensions 10 mm × 10 mm × 2.5 mm was printed via layer-by-layer deposition at the rate of 5 mm/s with a 27 G gauge syringe tip and extrusion pressure in the range of 47 to 80 kPa, depending on the bioink composition (50 ± 3, 76 ± 4, and 62 ± 2 kPa for compositions I, II, and III, respectively). The temperature of the print head was maintained in the range of 18–22 °C and the printing stage was kept at 10 °C to ensure smooth extrusion of the bioinks. Bioprinted scaffolds were crosslinked under the bioprinter-integrated UV lamp (365 nm) for 20 s, followed by incubation for 10 min in 2% *w*/*v* CaCl_2_ solution and washing in cell culture medium three times. Next, crosslinked scaffolds were transferred into a 6-well plate and incubated in the osteogenic medium at 37 °C and 5% CO_2_ for 21 days. The osteogenic medium was exchanged every three days.

It should be noted that due to the extremely low concentration of LAP photoinitiator in bioinks, only a limited degree of crosslinking is achieved via UV activation, and physical Ca^2+^-assisted crosslinking is needed as a complementary tool to achieve a stable and high-shape fidelity structure. Skipping the step of chemical crosslinking leads to early degradation of a scaffold, whereas omission of Ca^2+^ stitching leads to a rapid loss of shape fidelity and morphology, therefore, the latter step in hydrogel fixation was performed within the first few minutes after the printing.

### 2.8. Printing Accuracy

Printing accuracy (PA) is the difference between the actual dimensions of the CAD-designed model and the dimensions of the printed construct. PA was defined as a ratio between the sum of the theoretical and the actual surface area of the voids (A*_void_*) of the scaffolds [41] and expressed in percentage, Equation (2):

(2) $$\mathrm{Printing}\;\mathrm{accuracy}\;\%\;(\mathrm{PA})=\frac{\sum [A_{void}^{theor}-\sqrt{{(A}_{void}^{pract}}-A_{void}^{theor}{)}^{2}]}{\sum A_{void}^{theor}}\;\times \;100\%$$

The surface area of the void from the top view of the scaffolds was measured using ImageJ software. The area was calculated by taking an average of the four scaffolds with high precision and less than 5% variability among the tested scaffolds of each composition.

### 2.9. Scaffold Characterization

#### 2.9.1. Surface Morphology

The morphology of the printed scaffold was observed by Scanning Electron microscopy SEM (JSM-IT200, Jeol, Tokyos, Japan). The scaffolds were lyophilized and gold sputtering of 7 nm was applied before imaging. An accelerating voltage of 15 kV was used for visualization.

#### 2.9.2. Swelling and Degradation

Triplets of freeze-dried scaffolds of each composition were weighed before and after immersion in sterile PBS for 1 and 5 h at 5% CO_2_ and 37 °C. The weight of the swollen scaffold was measured after blotting the excess solution. The swelling capacity was calculated according to Equation (3):

(3) $$\mathrm{Swelling}\;\mathrm{capacity}\;\%\;(\mathrm{SC})=\frac{W(t)-W(0)}{W(0)}\times 100\%$$

where W(t) is the scaffold’s weight after each incubation time interval and W(0) is the dry weight of the scaffold.

For calculation of the degradation rate, first, freeze-dried scaffolds, W(0) were weighed and then placed in media consisting of DMEM with 10% FBS, and 1% penicillin-streptomycin for 14 days in the incubator at 5% CO_2_ and 37 °C. After the fixed periods, four scaffolds of each kind were taken out of the media, freeze-dried, and weighed. The degree of degradation was then calculated according to Equation (4):

(4) $$\mathrm{Degradation}\;\mathrm{degree}\%\;(\mathrm{DD})=\frac{W(0)-W(f)}{W(0)}\times 100\%$$

Since lyophilization after the degradation notably affects the geometry and general integrity of the scaffold, each scaffold was used only once for the degradation test. In each measurement, the average data of four replicates (N = 4) were used after the measurement.

#### 2.9.3. Compression Testing

The elastic modulus of the printed scaffolds was assessed using a texture analyzer (Stable Micro Systems, Godalming, Surrey, UK). To measure the scaffold’s compression resistance, tests were performed on the scaffolds after 1, 7, 14, and 21 days in PBS. The compression tests were carried out by subjecting the samples to up to 80% deformation using a 50 mm diameter plunger at a speed of 1 mm·s^−1^. The slope of the linear portion of the stress–strain curve between 10 and 20% strain was used to determine the compressive modulus (E) according to Equation (5):

(5) $$E=\frac{\frac{F}{A}}{\frac{∆h}{h}}$$

where *F* is the applied force, *A* is the scaffold’s area, Δ*h* is the change in height during compression, and *h* is the scaffold’s initial height. The measurements were conducted in triplicate (N = 3), and the averaged results are reported.

#### 2.9.4. Cell Viability and Morphology

Cell viability was evaluated on days 3, 7, and 14 by live/dead staining. The Calcein-AM (green fluorescence) stains for live cells and ethidium homodimer (red fluorescence) for dead cells. Briefly, after the culture media was aspirated on days 3, 7, and 14, scaffolds were incubated in staining media, which consisted of 2 μM calcein-AM and 4 μM ethidium homodimer at 37 °C and 5% CO_2_ for 40 min in the dark. The scaffolds were rinsed three times with PBS and then placed in imaging chambers (ibidi, μ-Slide) containing culture media. The imaging was conducted using a confocal laser scanning microscope LSM 780 (by ZEISS, Oberkochen, Germany) equipped with an ×20 objective lens. The calcein (live) fluorophore was excited at 404 nm, and its emission was detected at 517 nm, while the ethidium homodimer (dead) was excited at 517 nm and its emission was detected at 617 nm. Multiple representative images were captured from the scaffold at a magnification of ×20. The number of live and dead cells was determined by using ImageJ software (Fiji,v. 2.13.1), and the cell viability was calculated according to Equation (6), defined as the ratio between the number of live cells and the sum of live and dead cells:

(6) $$\mathrm{Cell}\;\mathrm{viability}\;(\%)=\frac{\#Live}{\#Live+\#Dead}\times 100\%$$

To improve visualization, color enhancement was utilized by maximizing the intensity.

#### 2.9.5. Osteogenic Differentiation ALP ELlSA

The osteogenic differentiation was observed on the bioprinted scaffold by evaluating the alkaline phosphatase (ALP) activity using the ALP ELISA kit (ab83369, Abcam, Cambridge, UK). Briefly, bioprinted scaffolds were incubated in osteogenic media without phenol red. The supernatants from the cultured scaffolds were collected on days 7, 14, 21, 28, 35, and 42 and were frozen until further use. The ALP activity was measured using the manufacturer’s protocol, where collected samples were transferred to a 96-well plate and diluted with assay buffer (1:1 ratio). The absorbance was measured on a microplate reader at OD 405 nm. The ALP activity was determined by a comparison between the measurement of the difference between the OD of the sample and the blank with a standard curve produced from an ALP standard solution (0–10 ng/mL).

#### 2.9.6. Histology

The scaffolds for the mineralization and ECM studies were fixated and cryo-embedded after 42 days. First, scaffolds were rinsed with 10 mM CaCl_2_ and 0.15 M NaCl solution. Next, the samples were fixed with 4% paraformaldehyde in 10 mM CaCl_2_ and 150 mM NaCl solution for 2 h at room temperature. After fixation, the scaffolds were rinsed twice with 10 mM CaCl_2_ and 150 mM NaCl solution and were immersed into a 10% sucrose with 10 mM CaCl_2_ in ultrapure water (UPW) for 2 h at room temperature followed by 16 h incubation in 30% sucrose with 10 mM CaCl_2_ in UPW at room temperature. The scaffolds were then frozen in cryomolds with OCT in liquid nitrogen and stored at −80 °C for posterior cryosectioning. The cryosectioning was performed using a cryotome (CryoStar NX70, Thermo Fisher Scientific, Waltham, MA, USA). Samples were mounted onto a sample holder by gluing with OCT and cut with a thickness ranging from 10 to 25 µm. Slices were mounted on silane pre-coated glass slides. After allowing the slices to dry in the air for 10 min, they were collected and kept at −20 °C for further use.

To determine the extent of calcium deposition in the scaffolds, Alizarin red S staining was performed. After OCT was removed by submerging slides in DI water for 2 min, the slides were immersed in alizarin solution (pH 4.2) for a duration of two minutes, followed by a dehydration process using a combination of acetone, acetone-xylene (in a 1:1 ratio), and xylene. The slides were mounted and covered with a DPX mounting medium and the cover glasses with dimensions 18 × 18 mm and 0.13–0.17 mm thickness.

Hematoxylin and Eosin (H&E) staining was carried out to visualize the ECM formation in the scaffolds over a period of 42 days. Following the washing step, the scaffolds were immersed in hematoxylin for two minutes, subsequently drained, and washed in tap water that was changed frequently for five minutes. Subsequently, the slices were stained with eosin for 1.5 min, then drained and dipped twice in containers of 95% and 100% ethanol for 20 s. Next, the samples were washed twice in xylene baths for five minutes, briefly dried, and then mounted with DPX and cover glasses. Imaging of the stained slides was performed on the inverted field microscope (Jenco Instruments, San Diego, CA, USA).

### 2.10. Statistics

The quantitative data were shown as mean values ± by standard deviation. To compare the data, ANOVA was employed, followed by Tukey’s test for conducting pairwise comparisons. Differences were considered statistically significant with a *p*-value less than 0.05. All data were analyzed using Origin software (2023, version 10.0.0.154).

## 3. Result and Discussion

### 3.1. Ink Formulation and Rheological Characteristics of the Alginate/Gelatin/GelMA Scaffold

Bioink composition is crucial in the development of high-fidelity and mechanically stable scaffolds [42,43]. As stated in Table 1, three Alginate/Gelatin/GelMA compositions of variable *v*/*w* ratio were identified as potential inks that satisfy the selection criteria of printability for 3D printed scaffold fabrication, and optimized, based on the preliminarily conducted screening. The choice of ingredient materials and their ratio were dictated by several factors, such as low cytotoxicity, commercial availability, and shear-thinning properties, suitable for 3D printing. Table S1 in Supplementary Materials demonstrates a grid of scaffold images produced from variable ink combinations and ratios. Printing conditions, such as temperature and extrusion speed were optimized during the primary screening, aiming to maximize the efficacy of uninterrupted extrusion and printing accuracy. The total polymer and crosslinking agent concentrations were tested and optimized based on the desired scaffold morphology and pore diameters (>100 µm), favorable to bone tissue formation. The candidate ink compositions that revealed decent printability and post-printing shape fidelity were considered and subjected to further investigation.

The selected compositions of Alginate/Gelatin/GelMA, namely (1%/4%/5%), (1%/8%/2.5%), and (1%/2%/10%) *w*/*v* were marked as I, II, and III, respectively for convenience. Whereas gelatin and GelMA provide the main matrix material allowing covalent crosslinking and granting mechanical robustness [44], alginate was used as an auxiliary component with excellent cytocompatibility that encourages cell growth [45], allowing an additional ionic crosslinking and thus, increased shape fidelity. During the initial screening of potential compositions, it was found that an increase in alginate above 1% *w*/*v* does not contribute to higher scaffold stability as a result of Ca^2+^ bridging, yet reduces the overall printability qualities of the inks. Thus, in all three compositions, alginate was kept constant at 1% *w*/*v*, while printability was regulated via gelatin to GelMA ratio and concentrations.

**Figure 1:**
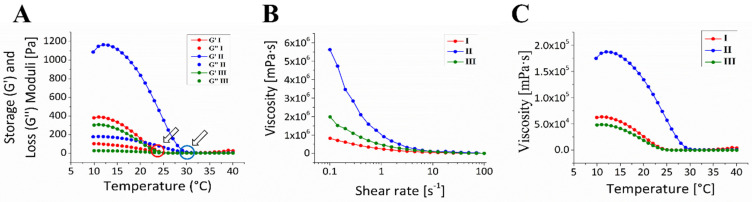
Rheological characterization of various (Alginate/Gelatin/GelMA) bioink compositions: I (1/4/5), II (1/8/2.5), III (1/2/10) in values of % *w*/*v*. (**A**) Storage (G′) and Loss (G″) moduli vs. temperature. Gelation points are circled and indicated with arrows, (**B**) Viscosity vs. shear rate at 10 °C, and (**C**) Viscosity vs. temperature.

The rheological properties of the inks were systematically examined for various temperatures and shear rate ranges. The gelation point was determined by calculating the storage and loss moduli (G′ and G″, respectively) as well as the change in viscosity in the range of relevant temperatures. Figure 1A depicts the viscoelastic characteristics of hydrogel compositions, with their respective gelation points, at G′ = G″. All compositions demonstrated a typical hydrogel behavior, with pronounced elastic behavior below gelation temperature, showing a decrease in storage and loss moduli with the elevation of temperature. Compositions I and III indicated a gelation transition at 24 °C, whereas the gelation point of composition II was observed at 29 °C. The higher gelation temperature of composition II, compared to the I and III is attributed to a higher content of gelatin as the main contributor to the ink viscosity. The lowest gelatin content in composition III was compensated by a high GelMA proportion, resulting in viscoelastic properties comparable to composition I. Figure 1B,C depict the viscosity of the inks at variable shear rates and temperatures, respectively. Shear thinning was observed in all compositions showing an inverse relationship of viscosity with increasing shear rate and temperature, providing a base for uniform extrusion of bioinks during extrusion. Due to the higher gelatin content, composition II is characterized by significantly higher viscosity, as compared to the other two inks (I and III). The higher viscosity suggests a higher shape fidelity and finer filament deposition, yet may lead also to lower cell survival due to a higher extrusion pressure during 3D printing. The optimal printing conditions were achieved at sub-solution/gel transition temperature (18 °C for compositions I and III, and 22 °C for composition II), at a low shear rate, to avoid cell damage, without compromising on printing accuracy and post-production shape fidelity.

### 3.2. Fabrication and Morphological Characteristics of 3D Bioprinted Scaffolds

**Scheme 1:**
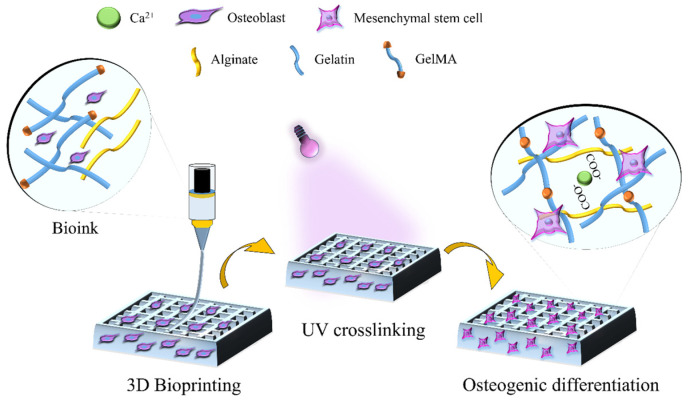
Fabrication of 3D printed Alg/Gel/GelMA-based cell-laden scaffolds.

The selected ink compositions were laden with mesenchymal stem cells and hydrogel scaffolds were successfully 3D printed, cured under 365 nm UV irradiations, and placed in a CaCl_2_ containing culture medium to facilitate covalent and ionic crosslinking, respectively, as depicted in Scheme 1.

**Figure 2:**
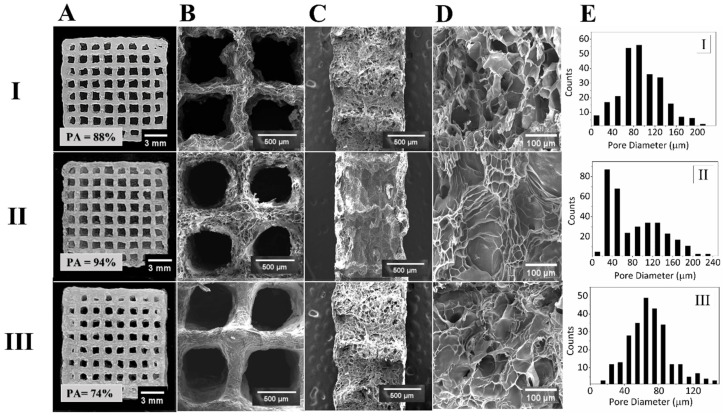
Optical images and obtained SEM micrographs of the various compositions (%Alginate/%Gelatin/%GelMA), I (1/4/5), II (1/8/2.5), III (1/2/10%); (**A**) Optical images of scaffolds after printing along with calculated printing fidelity, and SEM micrographs; (**B**) Top view; (**C**) Cross-sectional view; (**D**) Magnified SEM images of the inner region; (**E**) Distribution of pore sizes of the resulting 3D printed scaffolds I, II, and III.

Figure 2A demonstrates optical images of the resulting 15-layer-thick 3D-printed scaffolds, produced from the respective inks. Printing accuracy values are indicated in the image inset and correspond to 88%, 94%, and 74% for compositions I, II, and III, respectively. Due to the superior thermo-responsive characteristics of gelatin, the printing accuracy of ink composition II, with the highest content of gelatin among the three compositions revealed the highest values of accuracy and temporal shape fidelity. Although ink III exhibits higher viscosity values at low shear rates than ink I, owing to a greater GelMA concentration, the printing accuracy and shape fidelity of the scaffolds indicate a strong association with gelatin content in formulations.

The SEM micrographs, depicted in Figure 2B–D, correspond to the top, cross-sectional, and magnified views, respectively, indicating highly porous and interconnected structures for all scaffolds, produced from the corresponding ink compositions.

As a rule, a higher total polymer concentration or a high content of a crosslinking agent reduces the average pore size and increases pore density. Figure 2E demonstrates pore diameter profiles for the resulting 3D-printed scaffolds. Scaffolds I and III (produced from ink compositions I and III, respectively) revealed monomodal, normal pore size distribution profiles in the range of 20–200 µm, and 20–140 µm, respectively, with average pore sizes around 80 µm in both scaffolds. The highest total polymer concentration with the highest GelMA and the lowest Gel content in Scaffold III revealed the same average pore diameter as in Scaffold I but with a shorter size distribution profile at larger diameters. Unlike Scaffolds I and III, the pore size distribution of Scaffold II showed a bimodal profile with two main populations: smaller pores of ca. 40 µm and bigger cavities of ca. 120 µm average diameters. The morphology of Scaffold II can be described as an interconnected matrix with small pores branching off from the bigger antra. Such a bimodal morphology can be explained by a relatively high content of Gel and the resulting high viscosity of the ink, which hindered diffusion and led to the inhomogeneous distribution of methacrylate units, creating two pore size populations.

### 3.3. Physical and Mechanical Characterization of Alginate/Gelatin/GelMA Scaffolds

**Figure 3:**
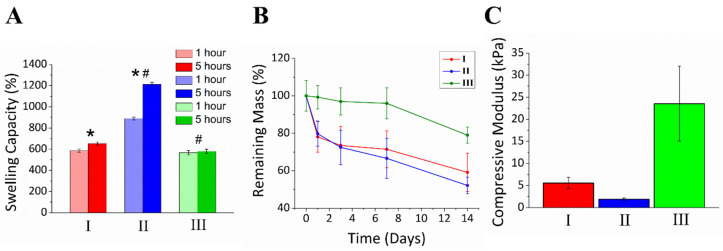
(**A**) Swelling capacity after 1 and 5 h in PBS. The symbols (* and #) indicate a statistically significant difference, based on *p* < 0.05 test, (**B**) Temporal degradation rates, and (**C**) Compressive moduli of the scaffolds produced from the respective ink compositions (I, II, and III) at 20% strain.

The physical and mechanical properties, namely swelling capacity, degradation rate, and stress/strain behavior of the produced scaffolds were evaluated. Figure 3A demonstrates the swelling capacity of the scaffolds after 1 and 5 h. Scaffold I demonstrated a swelling capacity of 585% after one hour, with a slight increase to 653 ± 5% after 5 h. Scaffold III revealed a very similar swelling capacity of only 580 ± 10%, which remained unaltered after 1 h and demonstrated a capillary effect, allowing the fast saturation of water molecules inside the scaffold. However, Scaffold II demonstrated superior swelling capacity, reaching ca. 900% after 1 h and 1213 ± 9% after 5 h. This behavior well correlates with the observed bimodal pore distribution, which suggests initial swelling of large pores, followed by detained swelling of the smaller cavities. The highest swelling capacity of Scaffold II also correlates with the lowest content of GelMA, responsible for the covalent hydrogel crosslinking, and the high gelatin ratio that provides the main building material. High polymer concentration allows the entanglement of water molecules inside the scaffold matrix, while lower crosslinking density contributes to the sharp wall thinning of the scaffold.

Figure 3B demonstrates the temporal degradation of the scaffolds in PBS over 14 days. Expectedly, the degradation rate of the scaffolds is inversely correlated with the water absorbance capabilities and GelMA content, i.e., the crosslinking degree. After 14 days, Scaffold III (10% *w*/*v* GelMA) retained ca. 80 ± 3.5% of its initial mass, whereas Scaffolds I (5% *w*/*v* GelMA) and II (2.5% *w*/*v* GelMA) revealed 59 ± 10.1% and 52 ± 3.8%, respectively. Due to the different degrees of crosslinking, Scaffold III shows a gradual degradation profile throughout the test, whereas Scaffolds I and II, demonstrate a major mass drop at day 1 from 100 to ca. 80%, followed by gradual degradation of ca. additional 20% after two weeks. As too rapid degradation indicates a weak hydrogel structure and a slower degradation rate may interrupt cell proliferation and extracellular matrix accumulation, the demonstrated degradation rates allow the use of hydrogel scaffolds in bone tissue engineering as cell-laden scaffolds. 

It should be noted that the demonstrated long-term stability of the scaffolds was achieved via a dual crosslinking approach, utilizing GelMA-based covalent and Ca^2+^-assisted ionic crosslinking. Aiming to increase cell viability, all ink compositions were infused with a minimal concentration of LAP photoinitiator (only 0.5% *w*/*v*), which was experimentally found effective in UV-initiated crosslinking. The exclusion of Alg from the recipe, or elimination of CaCl_2_ from the media dramatically decreased the mechanical stability of the scaffolds and negatively affects shape fidelity.

The compression test was performed to assess the mechanical characteristics of the resulting hydrogels. Figure 3C shows the values of the compressive modulus of the scaffolds at 20% strain. Compressive moduli profiles demonstrate correlation with GelMA content, placing the subjected scaffolds in the order of III > I > II from the stiffest to the most elastic construct, with values of 24 ± 8.5, 6 ± 1.3, and 2 ± 0.2 kPa, respectively. Figure S2 in Supplementary Materials shows the raw data of the mechanical stress/strain characterization of the studied scaffolds. In this test, four scaffolds of each type were subjected to mechanical deformation up to 45% of strain, from which compressive moduli (Young’s moduli) were calculated.

### 3.4. Cell Viability and Morphology

Efficient cell proliferation and successful colonization of the scaffold after the printing are crucial for its integration and ECM formation [46]. Mechanical properties of the scaffold can significantly impact these processes. It was revealed that having a stiffness >200 kPa improved the survival of cells over time and encouraged the development of a 3D cellular network [47].

**Figure 4:**
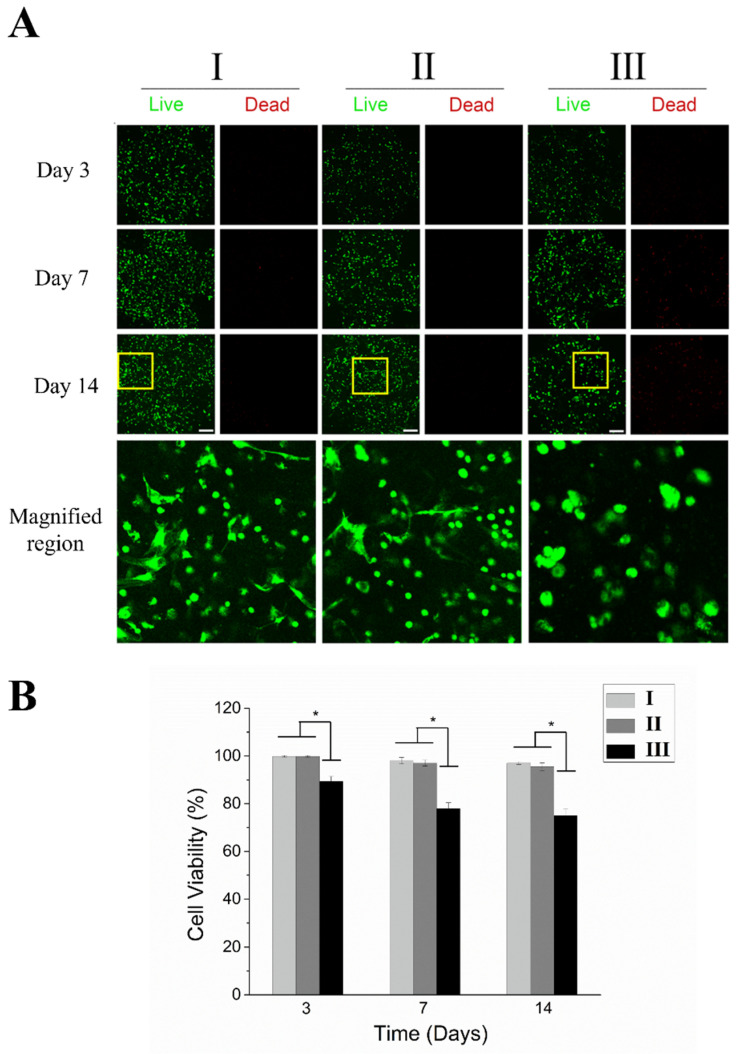
In vitro characterization of 3D bioprinted scaffolds with rMSCs. (**A**) The viability of the cells in the bioprinted scaffolds from the respective ink compositions (I, II, and III) was observed using live/dead staining on days 3, 7, and 14. Live cells were stained green and dead cells were stained red. The merged column shows live/dead staining overlayed with Hoechst. The images include magnified regions of individual stained cells, with color enhancement applied using Zen lite software (maximum intensity) for better visualization. The scale bar corresponds to a length of 100 µm. (**B**) A quantitative assessment of the cell viability within the scaffolds at days 3, 7, and 14. The symbols (*) indicate a statistically significant difference, based on the *p* < 0.05 test.

In the current study, we examined rat mesenchymal stem cell (rMSC) viability after 3D bioprinting and cell proliferation over 14 days of culturing in osteogenic media. Representative images of live and dead cells in 3D bioprinted cell-laden scaffolds on days 3, 7, and 14 are shown in Figure 4A. The predominance of green fluorescence indicates a higher number of alive cells, while a greater presence of red fluorescence indicates more dead cells. We qualitatively analyzed any changes in cell distribution and morphology over time in scaffolds with different compositions, Figure 4B. The cells in Scaffolds I and II had a minimum viability of 96%, with the highest viability observed after one day. There was no significant variation in the number of viable cells observed at different time intervals for both compositions, whereas Scaffold III showed significantly lower cell viability, revealing a continuously descending trend: 89.8 ± 1.9% on day 3, 75.1 ± 3.1% on day 7, and 72.6 ± 3.4% on day 14. Since Scaffold II was printed using the highest pressure among the studied ink compositions, yet revealed high cell viability, we attribute the low cell viability in Scaffold III to the excessive stiffness and higher crosslinking degree, rather than shear stress.

The magnified images in Figure 4A demonstrate the cell morphologies in the scaffolds on day 14. Most cells in Scaffold III retained spherical on all days. While in compositions I and II, cells exhibited spreading with a spindle-like morphology. We attribute the differences in cell viability and cell morphology exclusively to the composition of hydrogel bioinks. We conclude that despite demonstrating appropriate printability, high GelMA concentration leads to excessive crosslinking, which affects the physical properties of the scaffold, which in turn, hinders biological activity and cell functionality. The cell bioprinting was highly effective in compositions with a lower concentration of GelMA and Scaffolds I and II supported normal cellular metabolic function for a duration of several weeks. Jain et al. [48] reported that bioinks with lower GelMA density might promote better mass transfer, cell migration, and growth in softer environments compared to bioinks with higher GelMA density.

### 3.5. MSC Osteogenesis and Formation of Mineralized ECM within 3D Bioprinted Scaffolds

**Figure 5:**
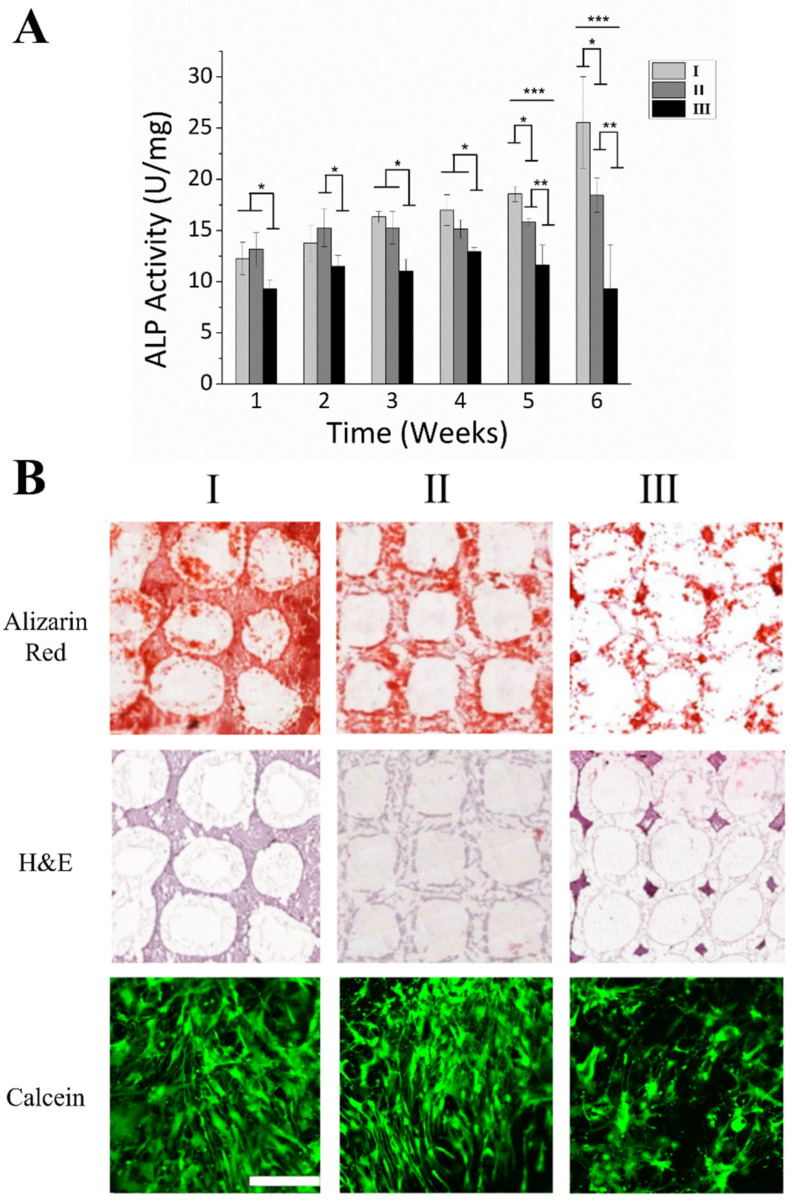
(**A**) ALP activity in uL/mg of the cells in the bioprinted scaffolds from the respective ink compositions (I, II, and III) within the 6 weeks. The symbols (*, **, and ***) indicate a statistically significant difference, based on the *p* < 0.05 test. (**B**). Light microscopy images of the histological stainings of the bioprinted scaffolds from the respective ink compositions (I, II, and III) after 6 weeks. Alizarin Red S staining indicates scaffold mineralization, results of H&E staining correspond to ECM formation and calcein staining depicts cell morphology within the scaffolds after 6 weeks. The scale bar corresponds to a length of 100 µm.

The differentiation of rMSCs into osteogenic cells was examined by analyzing alkaline phosphatase activity (ALP), indicating the presence of osteoblasts and, thus, new bone formation. Figure 5A displays the ALP activity of rMSCs in 3D bioprinted scaffolds over 6 weeks. Whereas ALP activity in Scaffolds I and II was significantly higher than in Scaffold III and continuously increased throughout 6 weeks; in Scaffold III, ALP activity demonstrated a decrease after week 4. After 6 weeks, Scaffold III showed only 8.5 ± 4.7 U/mg ALP activity, compared to 25.0 ± 4.6 and 17.5 ± 1.3 U/mg for Scaffolds I and II, respectively.

It is worth noting that on weeks 1 and 2, ALP activity in Scaffold I showed slightly lower values compared to Scaffold II, yet right after demonstrated rapid growth over 4 weeks, overtaking ALP activity in Scaffold II by ca. 30%.

The formation and mineralization of bone ECM within 3D bioprinted scaffolds were analyzed using histology after culturing over 6 weeks in osteogenic media, via Alizarin Red S, hematoxylin and eosin (H&E), and Calcein stainings, Figure 5B. The staining of Alizarin Red S indicated that the cell-laden Scaffold I exhibit a stronger red color intensity compared to Scaffolds II and III, suggesting that Scaffold I had a greater mineral content. Scaffold III revealed only minor and ragged color patterns due to the low mineral content. H&E staining showed a uniform cell distribution throughout Scaffolds I and II, while Scaffold III revealed only minor and non-uniform distribution, with staining mostly located in the knots of the scaffold’s voids. Calcein staining reveals the relationship between cell morphology, ECM formation, and mineralization after 6 weeks. Scaffolds I and II exhibited higher cell density, spindle-like morphology, and uniform spreading, in contrast to Scaffold III.

Previously, it was also shown that the osteogenic differentiation and ALP activity were increased for rMSCs cultured on stiffer 2D substrates [49]. However, our results show that in 3D materials cell proliferation and spreading can be hindered in stiff hydrogel matrices, which might reduce the potential for osteogenic differentiation.

The observed cell spreading morphology, as evidenced by cell images in Figure 4A and Figure 5B, may confer the potential to expedite the osteogenic differentiation of rMSCs in soft scaffolds and consequently ECM formation and mineralization.

The development of hydrogels through 3D printing necessitates two essential biomaterial properties. Firstly, the material should exhibit easy processability to enable the creation of high-resolution tissue-like structures. Secondly, it should be cytocompatible, thereby facilitating migration, proliferation, and differentiation of the embedded cells.

## 4. Conclusions

Aiming to increase viability in cell-laden 3D-printed scaffolds via minimizing photoinitiator concentration, we demonstrated the implementation of a dual-crosslinking approach on printable gelatin-based hydrogels of variable compositions. Rheological properties of three potential ink compositions comprising alginate, gelatin, and gelatin methacrylate were assessed and the respective cell-laden constructs were manufactured. Morphology, physical, and mechanical properties of the resulting scaffolds were studied and the biological activity of rat mesenchymal stem cells toward bone tissue formation and mineralization was examined for over 6 weeks. We showed that the biological activity of cells and tissue formation is inversely correlated with the stiffness of the produced scaffolds, which was controlled by the gelatin methacrylate component, as the main photocrosslinking agent. The dual-crosslinking approach implemented on Alg/Gel/GelMA inks allows using as low as 0.5% *w*/*v* of LAP photoinitiator, controlling inner and outer morphology of the fabricated scaffolds, compensating such low amounts with physical Ca^2+^-assisted stitching for extensive stability. Experimental data showed that ink formulation I (1% Alg/4% Gel/5% GelMA *w*/*v*) revealed a very impressive printing accuracy of ca. 90%, allowing the fabrication of high-resolution constructs characterized by macroporous matrix morphology and low cytotoxicity, which facilitates uniform cell spreading and unimpeded bone tissue development in vitro. Among the studied bioink compositions, the scaffold produced from ink III (1% Alg/2% Gel/10% GelMA) exhibited the lowest cell viability, and ECM deposition and mineralization as a result. A possible reason for that was a relatively high rigidity of the scaffold and significantly lower average pore size diameter. These are common factors that may affect cell proliferation and attachment. Moreover, the excessive rigidity manifests itself in the detained degradation, impeding the timely deposition of ECM and its structurization within the scaffold due to the space shortage.

The demonstrated approach implemented on optimized hydrogel ink formulation offers a simple yet effective technique for fabricating high-precision 3D-printed hydrogel scaffolds bone tissue engineering as well as other related applications.

## Figures and Tables

**Table 1:** Bioink compositions of alginate/gelatin/GelMA hydrogels.

Bioink Formulation	Alginate (mg/mL)	Gelatin (mg/mL)	GelMA (mg/mL)	PBS (mL)
I (1% Alg/4% Gel/5% GelMA)	100	400	500	8
II (1% Alg/8% Gel/2.5% GelMA)	100	800	250	8
III (1% Alg/2% Gel/10% GelMA)	100	200	1000	8

## Data Availability

Not applicable.

## Supplementary Materials

The following supporting information can be downloaded at: https://www.mdpi.com/article/10.3390/bioengineering10060704/s1, Figure S1: 1H Nuclear Magnetic Resonance spectra of gelatin and gelatin methacrylate (GelMA); Table S1: Optical images of scaffolds produced from variable gelatin/gelatin methacrylate (GelMA) ratios; Figure S2: Averaged stress-strain behavior of the scaffolds I, II, and III.
