# Supplementary material for: Dual-Crosslinking of Gelatin-Based Hydrogels: Promising Compositions for a 3D Printed Organotypic Bone Model
Source: Bioengineering (Basel). 2023 Jun 9;10(6):704. doi: 10.3390/bioengineering10060704 (PMC10295226; doi:10.3390/bioengineering10060704)
Supplement: Supplementary file 1 [file bioengineering-10-00704-s001.zip › bioengineering-2421054-supplementary.pdf]

bioengineering

MDPI

Supplementary Materials

Dual-Crosslinking of Gelatin-Based Hydrogels: Promising Compositions for a 3D Printed Organotypic Bone Model

Ahmer Shehzad, Fariza Mukasheva, Muhammad Moazzam, Dana Sultanova, Birzhan Abdikhan, Alexander Trifonov and Dana Akilbekova *

Department of Chemical Engineering, School of Engineering and Digital Sciences, Nazarbayev University, 010000 Astana, Kazakhstan

* Correspondence: dana.akilbekova@nu.edu.kz

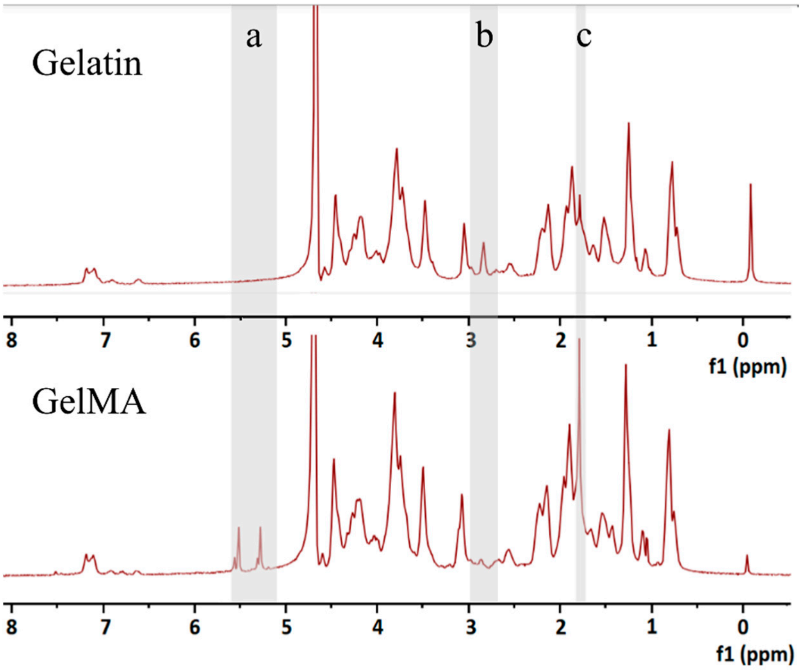

Figure S1. 1H Nuclear Magnetic Resonance spectra of gelatin and gelatin methacrylate (GelMA). The highlighted region: (**a**) 5.3 and 5.52 ppm correspond to the protons of the methacrylate vinyl group, (**b**) 2.82 ppm corresponds to the methylene of lysine's side chain, (**c**) 1.75 ppm corresponds to the substitution of amino groups by methyl protons of methacryloyl.

Table S1. Optical images of variable gelatin/gelatin methacrylate (GelMA) ratios. Representable printing fidelity (PF) of the scaffolds 10 minutes after the printing.

**Gelatin w/v%**	**GelMA w/v%**		
	**2.5**	**5**	**10**
0	Not Printable	Not Printable	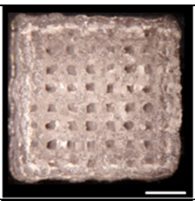
2	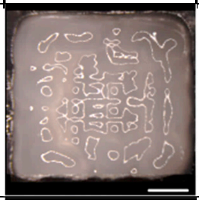	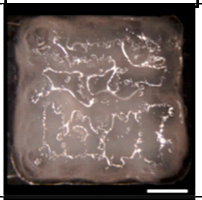	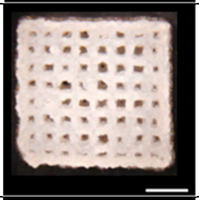
4	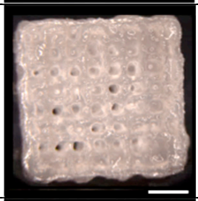	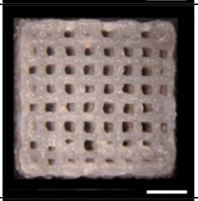	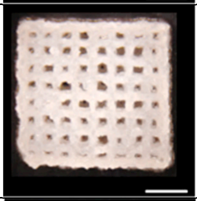
6	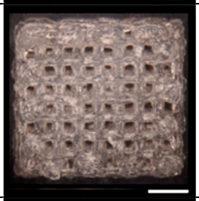	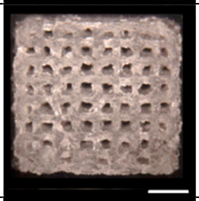	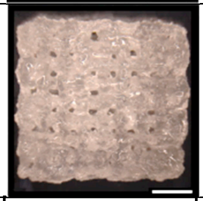
8	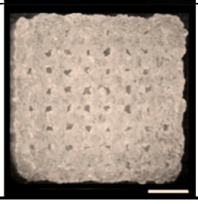	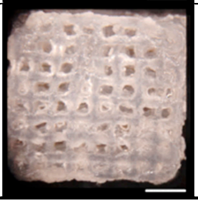	Not Printable

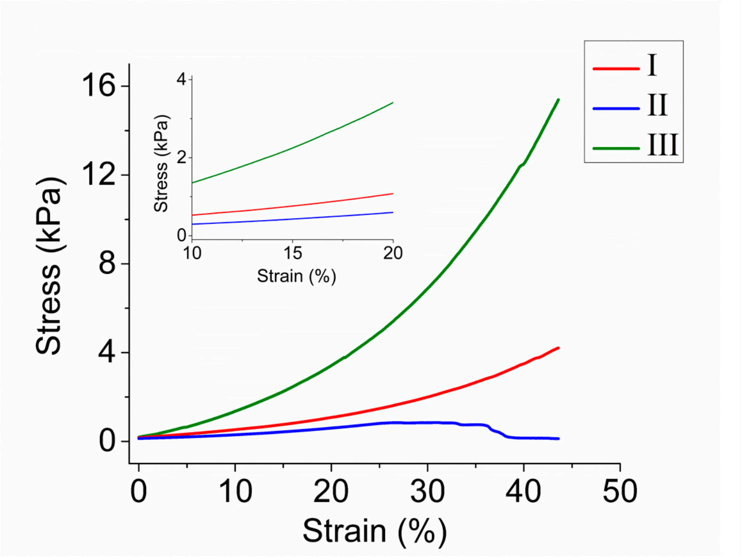

Figure S2. Averaged stress-strain behavior of the respective scaffolds (I, II, and III). All scaffolds were subjected to a strain of up to 44%. Inset: magnified region, from which compressive moduli were calculated. N = 4 for each scaffold.
